# First Presentation of Endometriosis: A Case of Acute Large Bowel Obstruction

**DOI:** 10.7759/cureus.91306

**Published:** 2025-08-30

**Authors:** Aiah Al-Saig, Lakshmy Nandakumar, Jane Theodore, Jayson Moloney

**Affiliations:** 1 Colorectal Unit, Department of General Surgery and Burns, Royal Brisbane and Women’s Hospital, Brisbane, AUS; 2 Anatomical Pathology, Pathology Queensland, Brisbane, AUS

**Keywords:** acute bowel obstruction, bowel endometriosis, endometriosis, endometriosis diagnosis, hartmann’s procedure

## Abstract

The first presentation of endometriosis as acute large bowel obstruction is rare. We present the case of a woman in her 40s with no significant past medical history presenting with acute bowel obstruction. CT imaging revealed an obstructing mass in the sigmoid colon with associated lymphadenopathy, favoured to be a malignant process. She underwent an emergency laparoscopic to open Hartmann’s procedure, where the sigmoid mass was seen causing proximal colonic distention and caecal seromuscular tears. Her case was discussed at the colorectal multidisciplinary team meeting, where pathology revealed a surprising diagnosis of endometriosis; no primary mucosal lesion was identified. This case highlights endometriosis as an important differential cause of acute bowel obstruction, even in patients with no prior history of endometriosis.

## Introduction

Endometriosis is a chronic, estrogen-dependent inflammatory disorder characterised by the presence of endometrial tissue outside the uterine cavity and whose pathophysiology remains poorly understood [1]. It affects around 10% of women of reproductive age and is typically associated with pelvic pain, dysmenorrhea, dyspareunia, and infertility [2]. Endometriosis can be broadly categorised into the following three groups: superficial peritoneal lesions, cystic ovarian lesions, and deep sub-peritoneal lesions, also known as deep infiltrating endometriosis (DIE), with up to 12% of these involving the gastrointestinal tract [3]. Bowel endometriosis often presents with non-specific or cyclical gastrointestinal symptoms such as bloating, constipation, or rectal bleeding. True bowel obstruction is a rare complication, accounting for fewer than 1% of all cases [4]. Obstruction typically results from transmural infiltration of ectopic endometrial tissue into the muscularis propria and serosa, leading to fibrosis, smooth muscle hyperplasia, and stricture formation [5]. Mucosal involvement is rare, and superficial endoscopic biopsies often fail to accurately diagnose and stage disease, complicating preoperative diagnosis.

Radiological investigations for DIE involving the bowel may show non-specific findings such as bowel wall thickening or mass effect; however, MRI, particularly with bowel preparation and T2-weighted sequences, has been shown to be more sensitive in detecting DIE [6]. Despite modern advancements in medical imaging technologies, intestinal endometriosis is frequently misdiagnosed as Crohn’s disease, colorectal carcinoma, or other more common causes of bowel obstruction.

Acute large bowel obstruction secondary to endometriosis is often challenging to diagnose preoperatively and presents a complex management dilemma. Partial or subacute obstruction can theoretically be managed with hormonal suppression, such as gonadotropin-releasing hormone agonists, to relieve symptoms [7]. However, in cases of progressive or complete obstruction, such as in our case, bowel resection is often required. The type of bowel resection can vary depending on clinical urgency and can range from segmental colectomy and anterior resection to emergent Hartmann’s procedure or ileocolic resection. Hence, this rare but serious manifestation of endometriosis reminds surgeons of the need to maintain a broad differential for causes of acute large bowel obstruction, especially in female patients of reproductive age. In particular, this case highlights that an absence of endometriosis history does not exclude this diagnosis. A multidisciplinary team (MDT) approach involving colorectal surgeons, gynaecologists, pathologists, and radiologists is essential for effective diagnosis and management.

## Case presentation

A woman in her 40s presented to the Emergency Department with a two-day history of worsening cramping lower abdominal pain. She denied a history of nausea, vomiting, or diarrhoea and rated the pain 9 out of 10 on a subjective pain scale. She had not opened her bowels for three days and reported usually having once-daily regular bowel motions.

She had no past medical or surgical history that she was aware of, was nulliparous, and did not take any regular medications. She reported having a regular menstrual cycle once a month and denied any history of dysmenorrhea, dyspareunia, or abnormal bleeding. Family history was significant for colorectal cancer (paternal grandfather).

On examination, her abdomen was mildly distended but soft, and she was tender in the suprapubic and lower quadrants bilaterally. There were no signs of peritonism, and bowel sounds were present. Her vital signs were unremarkable. Initial investigations, including basic blood findings shown in Table 1, were unremarkable aside from a mildly raised white blood cell count of 11.80 × 10^9^/L. Importantly, her carcinoembryonic antigen level was normal at 1.2 µg/L.

**Table 1:** Laboratory values from blood taken on the day of presentation.

Investigation	Units	Value	Reference range
Haemoglobin	g/L	139	115–160
White blood cell count	×10^9^/L	11.80	4.0–11.0
Sodium	mmol/L	140	135–145
Potassium	mmol/L	3.80	3.50–5.20
Chloride	mmol/L	106	95–110
Magnesium	mmol/L	0.90	0.70–1.10
Phosphate	mmol/L	1.26	0.75–1.50
Calcium	mmol/L	2.45	2.10–2.60
Creatinine	µmol/L	86	45–90
Lactate	mmol/L	1.10	0.50–2.20
Carcinembryonic antigen	µg/L	1.20	<3.50

**Figure 1:**
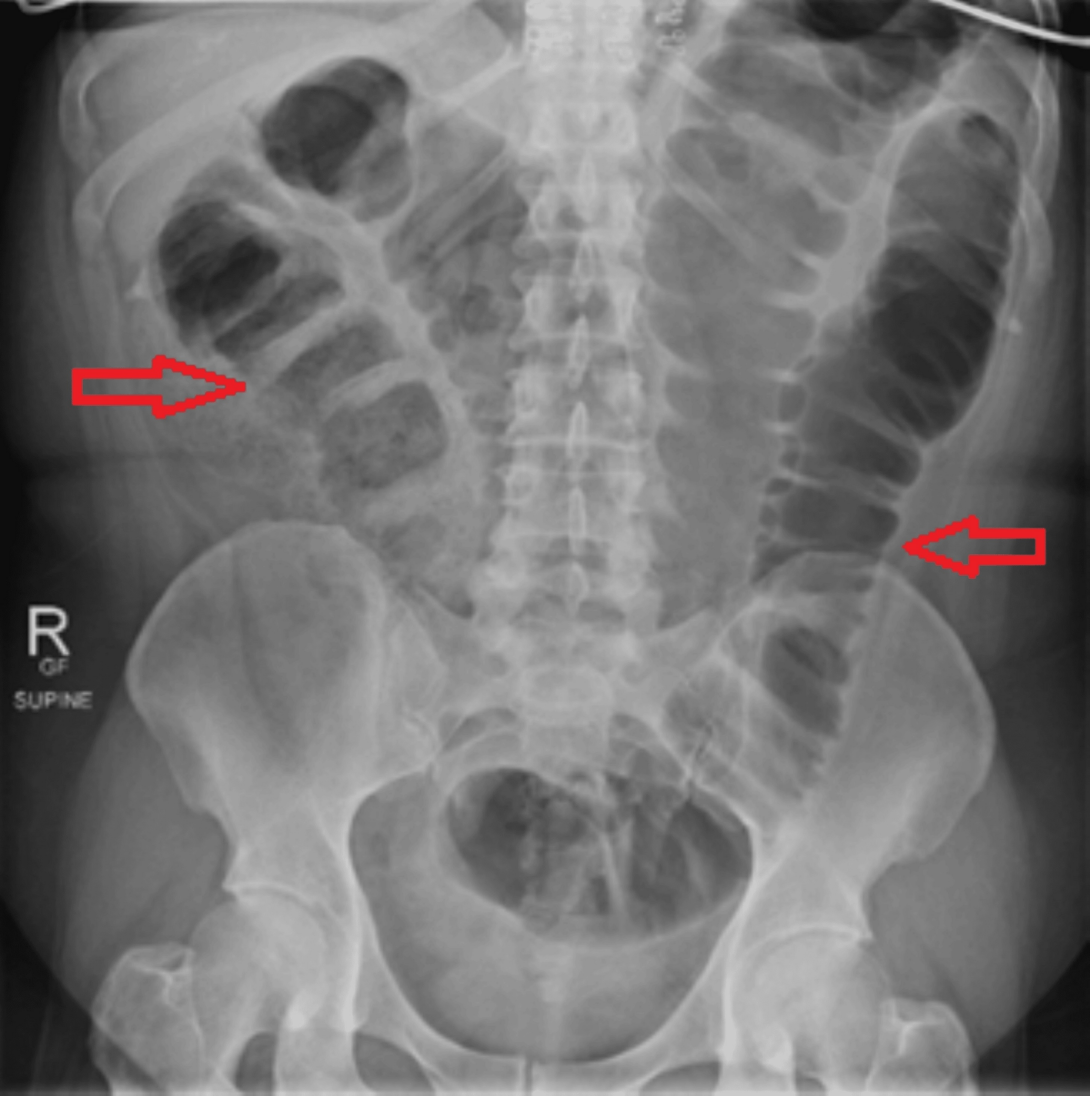
Abdomen X-ray showing distended large bowel loops (red arrows).

**Figure 2:**
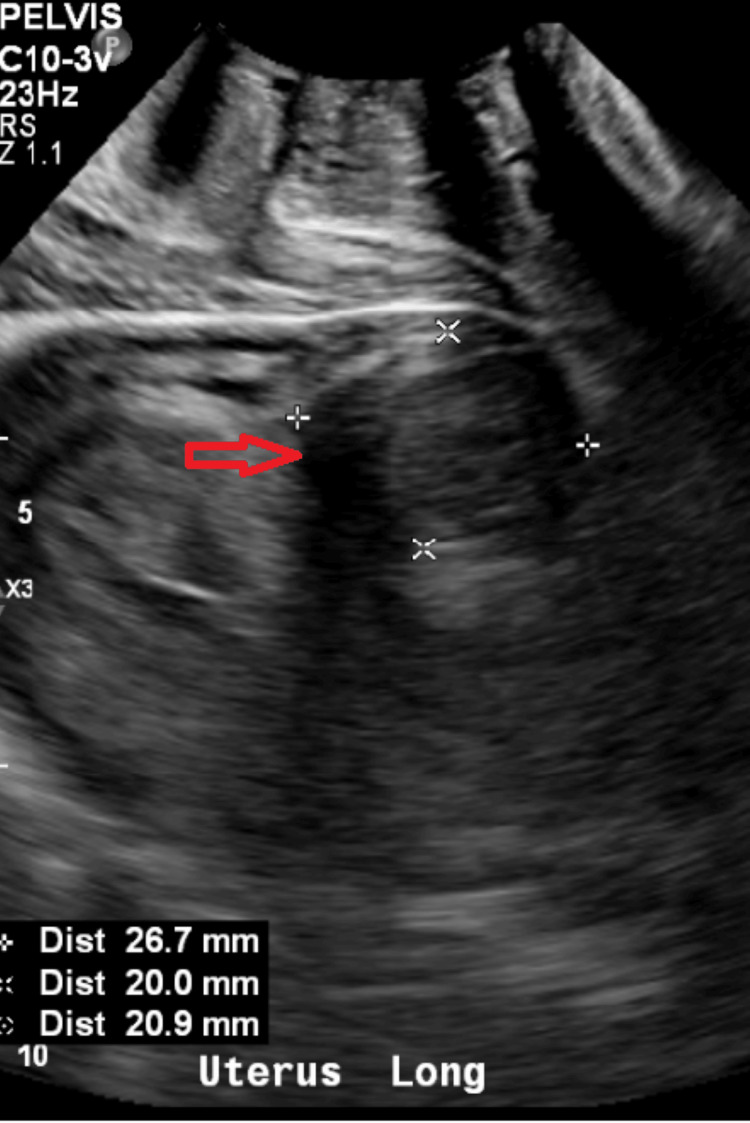
Pelvic ultrasound scan demonstrating uterus with a bulky fibroid measuring 27 × 20 × 21 mm (red arrow).

**Figure 3:**
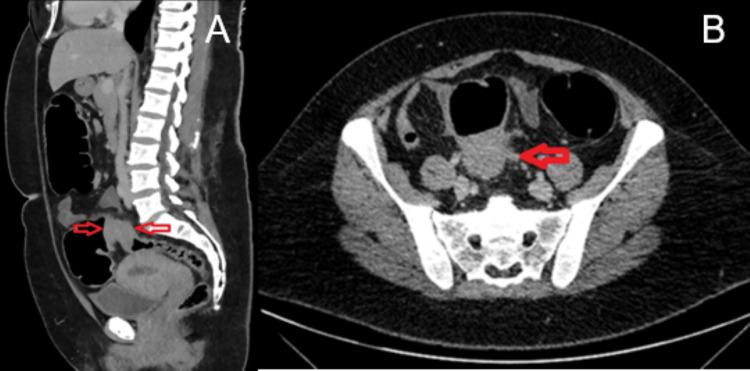
Sagittal CT scan of the abdomen and pelvis (A) demonstrating an obstructing mass at the sigmoid colon with thickening of the bowel wall and irregularity of the serosal surface (red arrows). Axial CT scan of the abdomen and pelvis (B) demonstrating small nodules within the adjacent mesentery of the sigmoid colon (red arrow).

**Figure 4:**
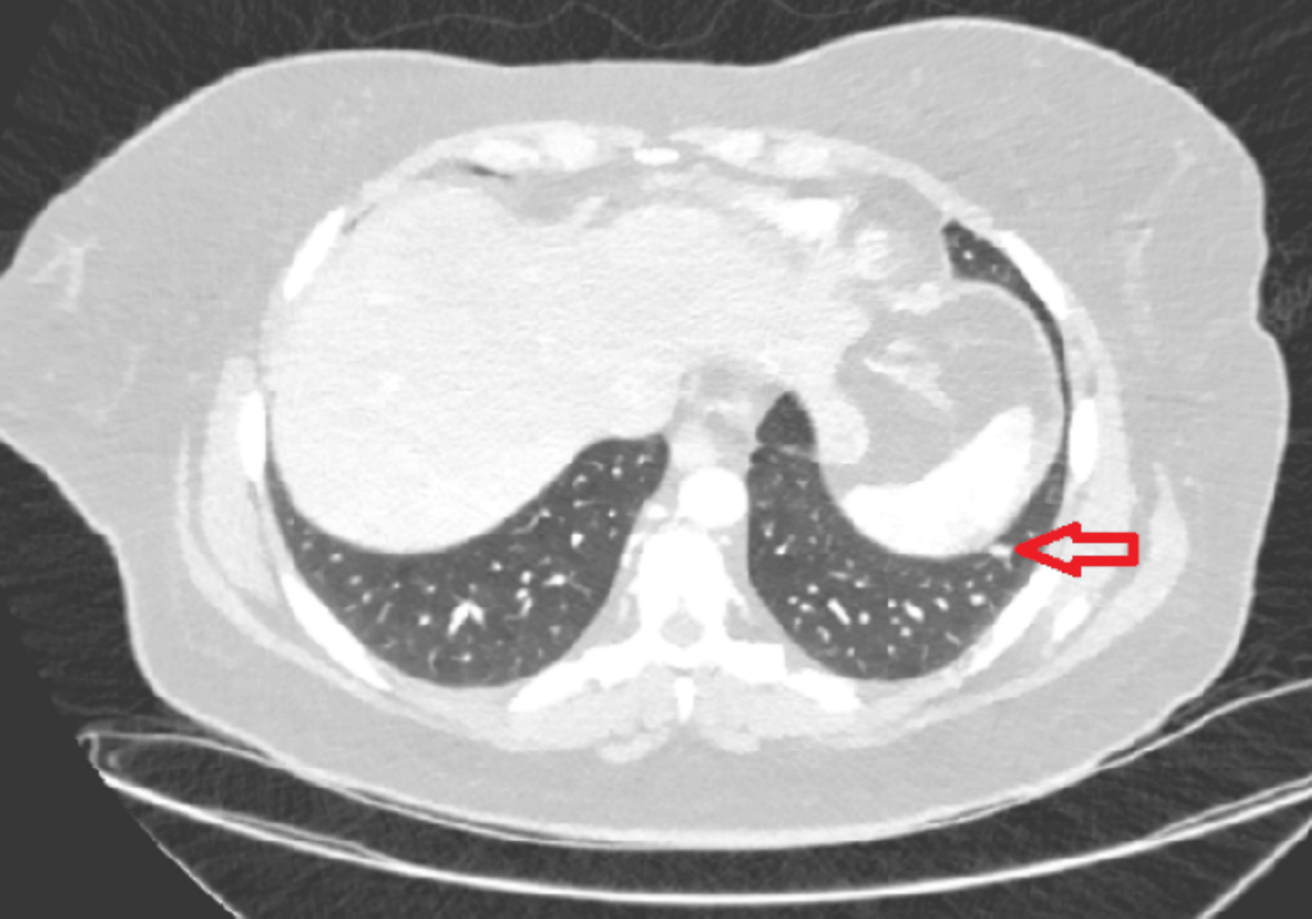
Axial CT scan of the chest demonstrating a small subpleural nodule measuring 5-6 mm in the left lower lobe (red arrow).

Initial imaging with plain radiograph of the abdomen (Figure 1) showed dilated large bowel loops, and an ultrasound scan of the pelvis showed an incidental intramural uterine fibroid (Figure 2) but otherwise normal appearing ovaries and pelvis. A CT scan of the abdomen and pelvis was performed (Figure 3) to assess for mechanical bowel obstruction, and an obstructing mass in the sigmoid colon was identified with associated lymphadenopathy. At this stage, the primary differential diagnosis was a primary colonic malignancy, and the patient underwent a staging chest CT scan, which showed several 5-6 mm pulmonary and subpulmonary nodules, an example of which is shown in Figure 4.

The patient now had a confirmed mechanical bowel obstruction, which was suggested to be secondary to a malignant process, and she was referred to the colorectal surgery team. The initial management plan included performing a flexible sigmoidoscopy and stenting as a bridge to definitive management with elective anterior resection. The patient was placed on intravenous fluid therapy, made nil by mouth and charted a Fleet enema, after which she had a very small bowel motion.

**Figure 5:**
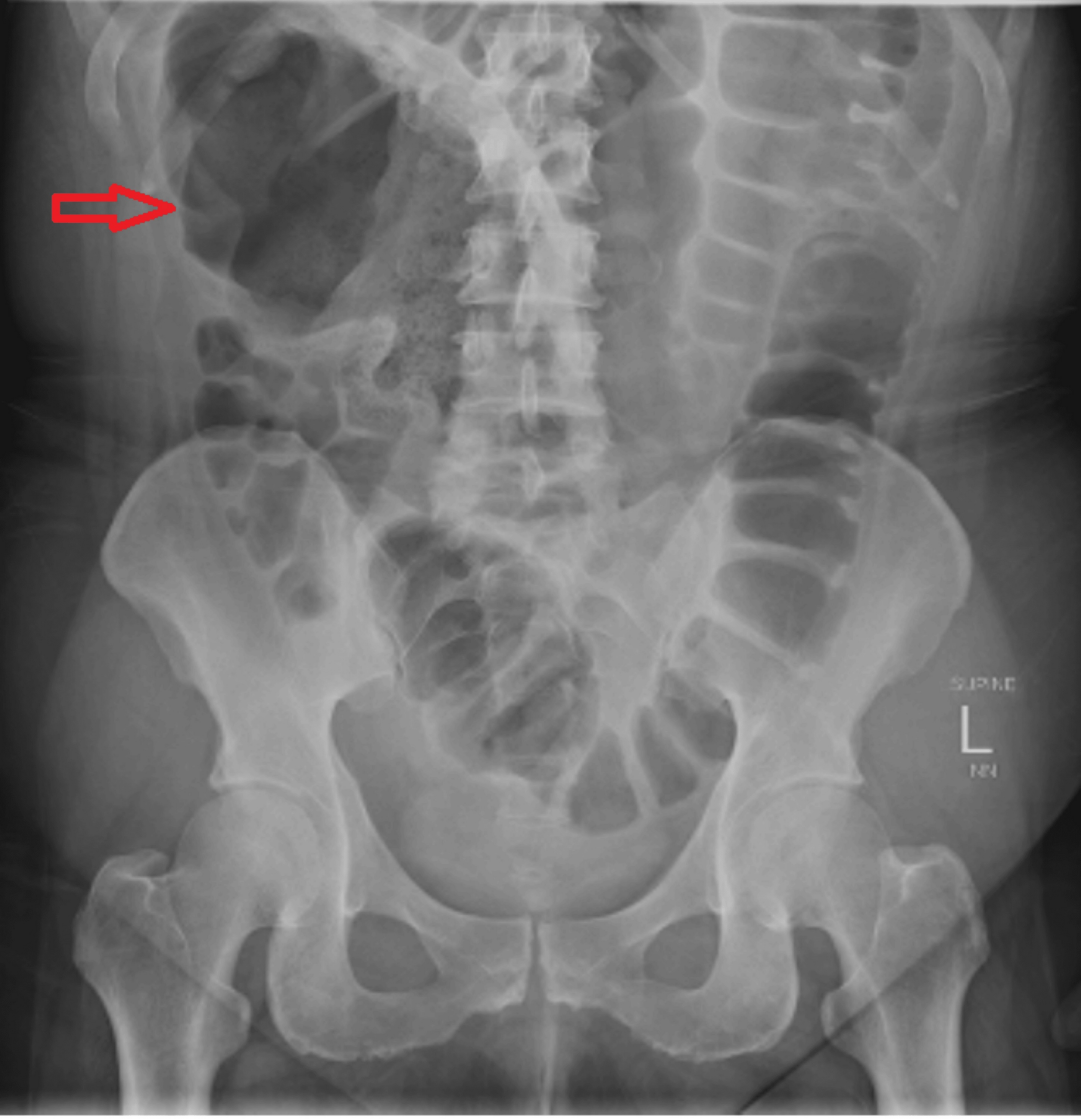
Abdominal X-ray demonstrating increasing colonic dilation (red arrow).

Due to clinical deterioration, a second plain abdominal radiograph (Figure 5) was performed approximately 24 hours later, which revealed a progressively dilated caecum, prompting an emergent laparoscopic to open Hartmann’s procedure. A flexible sigmoidoscopy performed intraoperatively confirmed a sigmoid mass 25 cm from the anal verge extending proximally to the point of obstruction, which was not traversable. There was gross colonic distension to the palpable obstructing sigmoid mass, and three large seromuscular tears in the caecum were identified, with a small volume of serous ascites present. Given the depth of the tears and risk of perforation, an ileocolic resection was also performed. No obvious peritoneal or visceral disease was visualised. The resected tissue was sent for histology, and the patient’s case was referred to the colorectal MDT for discussion. The patient had an uneventful postoperative recovery and was discharged after undergoing stoma education to facilitate independent management of her stoma at home, with follow-up appointments arranged for two and six weeks post-discharge date.

**Figure 6:**
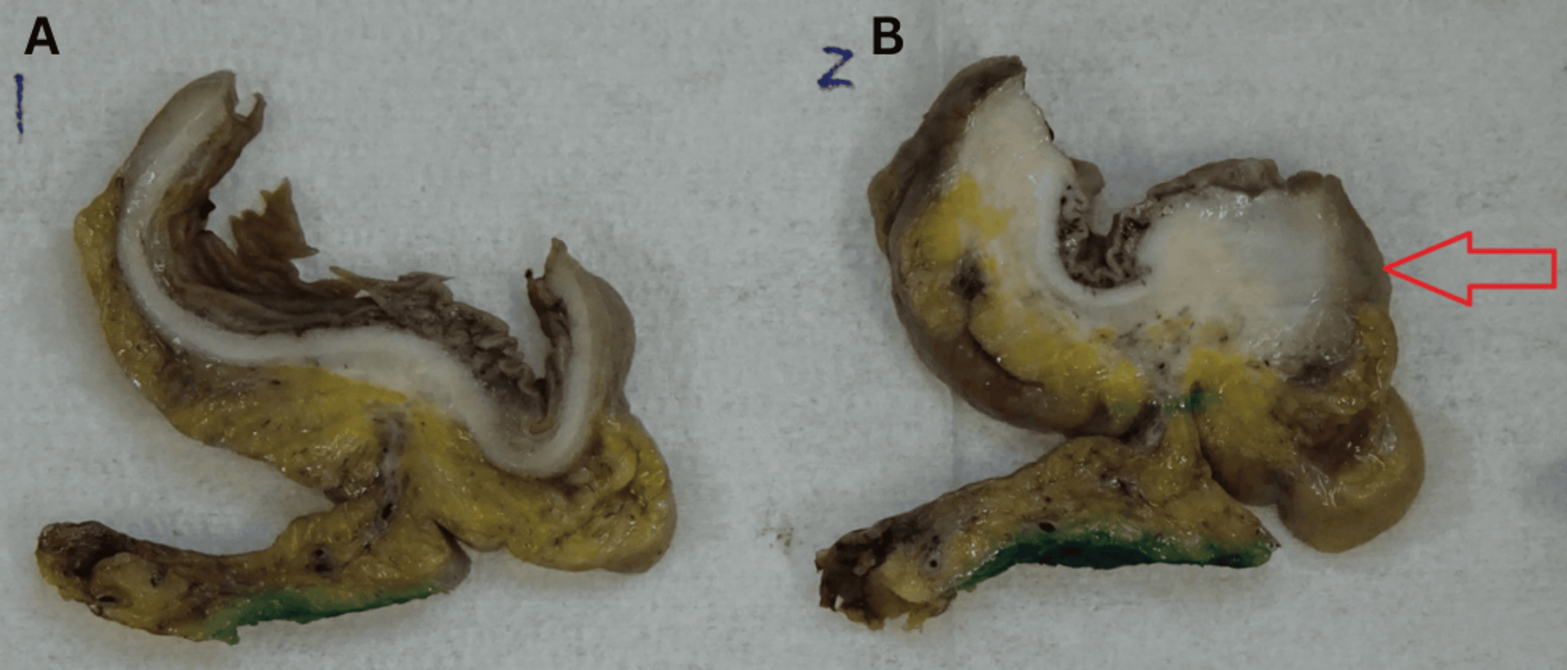
Macroscopic slices of sigmoid colon showing normal bowel (A) and a tumour-like mass of the thickened bowel indicated by a red arrow (B).

**Figure 7:**
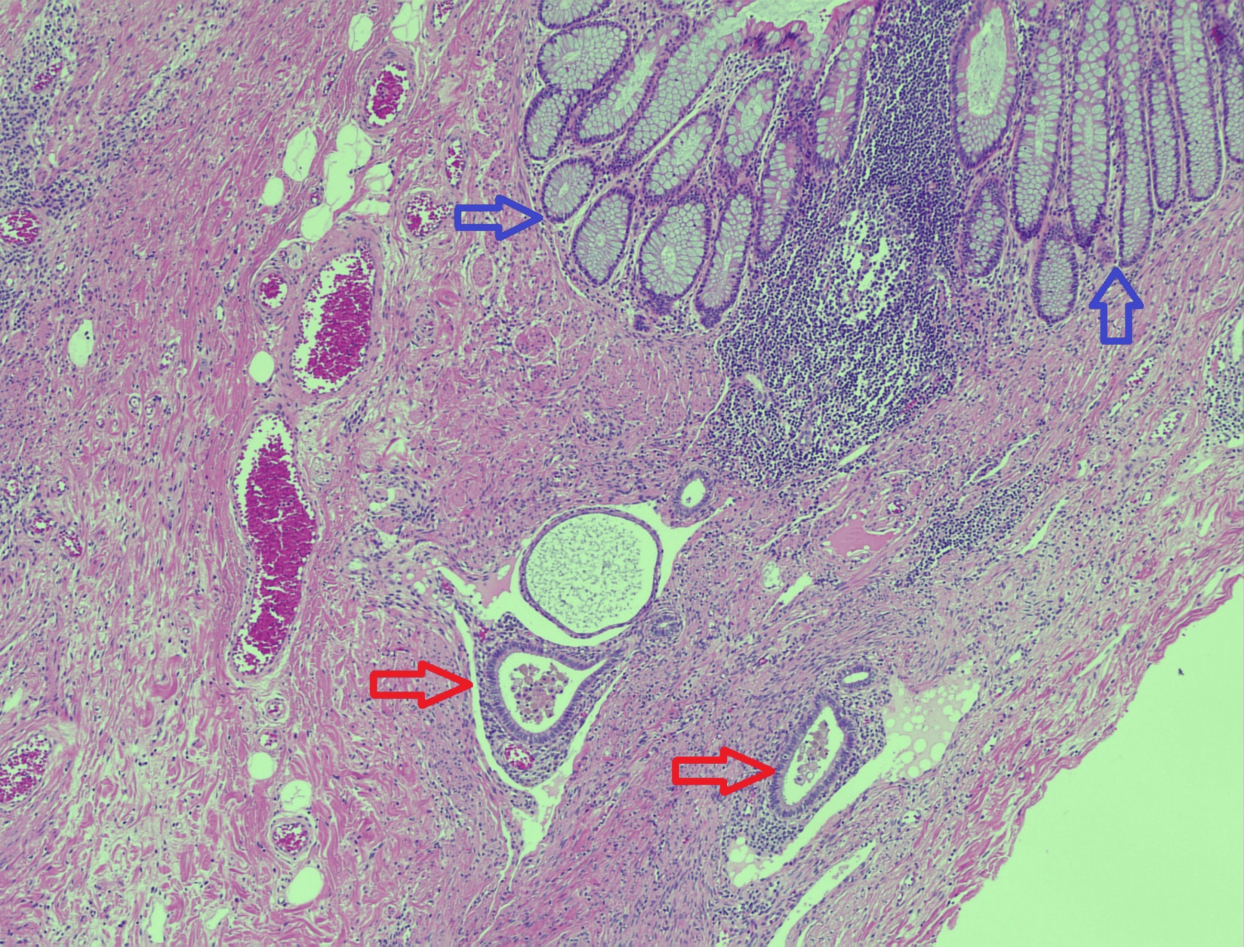
Micrograph showing endometrial-type glands and stroma (red arrows) with hemosiderin-laden macrophages in the gland lumen and normal overlying colonic mucosa (blue arrows) (hematoxylin and eosin, ×5).

**Figure 8:**
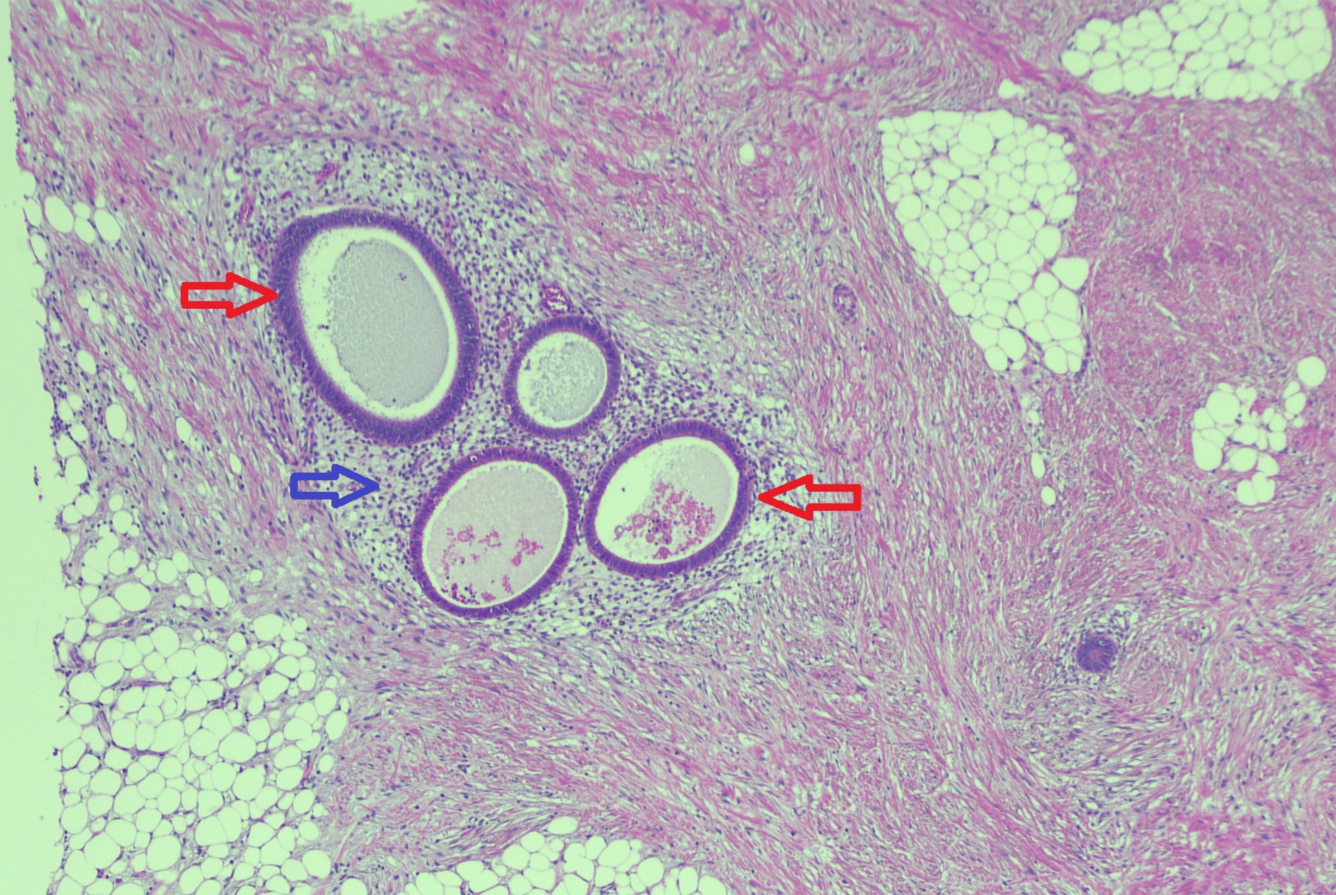
Micrograph showing endometrial glands (red arrows) and stroma (blue arrow) in subserosa with associated fibrosis (hematoxylin and eosin, ×5).

The MDT discussion revealed a surprising diagnosis of deep infiltrating endometriosis. Macroscopic findings showed a 38 × 38 × 30 mm tumour-like mass with undulating mucosa and thickened bowel wall in the sigmoid colon (Figure 6). Micrograph findings revealed endometrial-type glands and stroma in the submucosa, muscularis propria (Figure 7), and in the subserosa of the colon (Figure 8) with associated fibrosis and normal overlying mucosa.

**Figure 9:**
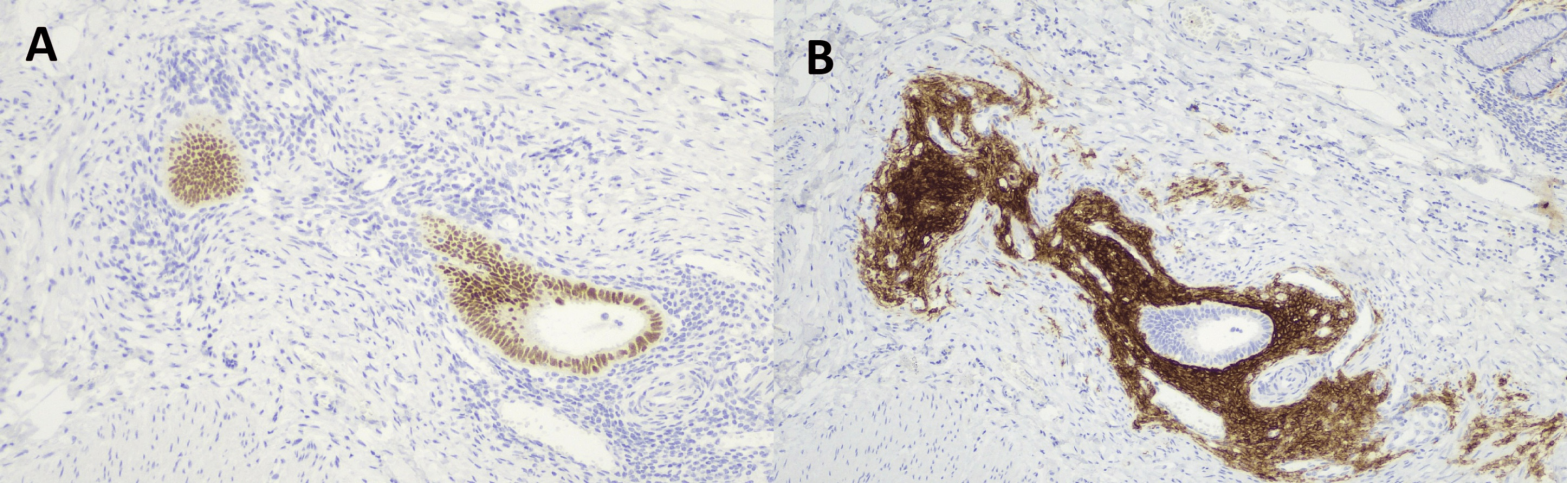
Endometrial glandular epithelium is PAX8 positive (×5) (A) and endometrial stromal cells are CD10 positive (×5) (B).

Specialised staining was undertaken to confirm endometrial glandular epithelium (Figure 9A) and endometrial stroma cells (Figure 9B). Of note, 23 benign reactive lymph nodes were seen measuring up to 10 mm. The patient was informed of the results and is planned for a Hartmann’s reversal procedure six months from her initial surgery date.

## Discussion

Endometriosis is an enigmatic disease that can present a diagnostic and management dilemma. Although the pathophysiology of this systemic disease is not well understood, several theories have been proposed in the current literature. One of the leading theories is that of retrograde menstruation, whereby viable endometrial tissue in menstrual fluid is refluxed back through the fallopian tubes into the peritoneal cavity to implant onto pelvic organs [2]. Other hypothesised theories include coelomic metaplasia and lymphatic and vascular metastasis, particularly in the case of endometriosis located outside of the pelvis [8]. To add to this complexity, endometriosis symptoms can range from completely debilitating to asymptomatic. Most typically, patients present with a history of pelvic pain, dysmenorrhoea, dyspareunia, and/or infertility, although oftentimes history can be less specific, e.g. chronic fatigue [2].

Bowel obstruction is an uncommon but well-documented complication of endometriosis, with most cases in the literature describing patients with known endometriosis or longstanding symptoms presenting with small or large bowel obstruction [4-5,9-11]. Bowel endometriosis can occur at any point along the gastrointestinal tract but has a predilection for the rectum and sigmoid colon [12]. This ectopic endometrial tissue on the bowel can be superficial (limited to the peritoneal and serosal layers) or deep disease, i.e. DIE (extending into the muscular and submucosa layers), with the latter sometimes invading into the bowel lumen [12]. The mechanism leading to bowel obstruction is thought to involve the response of the bowel’s smooth muscle to inflammation from ectopic endometrial tissue, generating extensive fibrosis that can ultimately result in bowel obstruction [9].

However, bowel obstruction as an initial presentation of endometriosis is a very rare occurrence, with only two other cases reported in the literature [13-14]. One of these cases presented with partial bowel obstruction and was found to have endometriosis of the sigmoid colon, and the other presented with acute obstruction, like our patient, and was eventually diagnosed with endometriosis of the rectum. As with our case, both underwent upfront colonic resection as the obstructing segment was impassable with an adult colonoscope, and both were only diagnosed with endometriosis upon the return of histopathology results; that is to say that patient history, preoperative imaging, and intraoperative findings were not suggestive of endometriosis. Delayed diagnosis of endometriosis is common even in symptomatic patients; therefore, in asymptomatic patients presenting with an unusual complication such as acute bowel obstruction, this broad differential is rarely entertained. Rather, surgeons maintain a high index of suspicion of a malignant process, particularly when imaging reveals a colonic mass with associated lymphadenopathy, as in our case. Furthermore, an incidental finding of pulmonary nodules, given the clinical context, further influenced clinical gestalt towards a malignant diagnosis. Increasingly, younger patients are presenting with late signs of colorectal cancer such as large bowel obstruction, possibly contributing to this cognitive bias [13-15]. These factors favoured an oncological approach to management, i.e. resection of local lymph node groups and referral to colorectal cancer MDT. Of note, serum levels of the tumour marker carcinoembryonic antigen (CEA) are high in approximately 70% of colorectal cancer patients at the time of diagnosis, with a negative predictive value (NPV) of above 80% [16]. This is important to consider when interpreting the normal CEA seen in our case and indeed other similar reported cases [13-14]; although the high NPV of this tumour marker makes a diagnosis of colorectal cancer less likely, almost one-third of colorectal cancers have a normal CEA level [15,16]. Therefore, interpreting the CEA level alongside the clinical context of each case is critical for sound clinical reasoning.

Interestingly, one of the cases similar to ours also underwent postoperative medical treatment with Goserelin, a luteinising hormone-releasing hormone analogue, to treat asymptomatic residual endometriomas near the resected bowel margins seen on MRI three months after resection, with reportedly good evolution on MRI after three months of therapy [15]. Medical management of endometriosis includes non-hormonal therapy such as non-steroidal anti-inflammatory medications, sometimes alongside hormonal therapy such as the oral contraceptive pill or gonadotropin-releasing hormone analogue, e.g. Goserelin, depending on local guidelines [8]. However, in the case of bowel endometriosis, typically aggressive upfront surgery in the first instance improves symptoms and quality of life, as in our case and others [3,7].

In this unique case, patient history, preoperative imaging, and intraoperative findings did not point to endometriosis as a cause of acute bowel obstruction. Evidently, preoperative diagnosis remains challenging; hence, laparoscopy is the gold standard tool for diagnosis [2]. However, in certain cases, intraoperative findings are also not suggestive of endometriosis, as in our case, no ectopic endometrial deposits or haemorrhage were visualised, and the obstructing sigmoid mass was hard and mostly fibrotic. It was microscopic histopathology alone that revealed the surprising diagnosis, including specialised staining to confirm endometrial glandular epithelium and stromal cells within the bowel wall. From this case, it can be learnt that, although uncommon, endometriosis must be considered a differential diagnosis in the female patient of child-bearing age presenting with signs and symptoms of bowel obstruction, and that having no prior history of endometriosis does not exclude severe disease.

## Conclusions

Endometriosis is a disease of variable presentation. This case demonstrates that an absence of endometriosis history does not exclude severe disease, and surgeons should be aware of this as a cause of acute bowel obstruction in young female patients to avoid unnecessary morbidity in the form of bowel resection and stoma formation.

## References

[1] (2025). World Health Organization. Endometriosis. https://www.who.int/news-room/fact-sheets/detail/endometriosis.

[2] Zondervan KT, Becker CM, Missmer SA (2020). Endometriosis. N Engl J Med.

[3] Remorgida V, Ferrero S, Fulcheri E, Ragni N, Martin DC (2007). Bowel endometriosis: presentation, diagnosis, and treatment. Obstet Gynecol Surv.

[4] Kutluk F, Ergün S, Uludağ SS (2025). Intestinal endometriosis: a rare cause of acute care surgery. Ulus Travma Acil Cerrahi Derg.

[5] Santos-Manzur A, Valdez-Bocanegra DR, Ornelas-Flores MC, Pineda-Díaz J, Stoopen-Margain E (2020). Ileal obstruction caused by transmural endometriosis in a patient with simultaneous C. difficile colitis and Influenza AH1N1. Case report. Int J Surg Case Rep.

[6] Minordi LM, Larosa L, Barbaro B (2023). How the radiologist must reason for a correct diagnosis in patients with small bowel mural thickening studied by CT or MRI: a pictorial review. Curr Probl Diagn Radiol.

[7] Rafique S, Decherney AH (2017). Medical management of endometriosis. Clin Obstet Gynecol.

[8] Horne AW, Missmer SA (2022). Pathophysiology, diagnosis, and management of endometriosis. BMJ.

[9] Mușat F, Păduraru DN, Bolocan A, Constantinescu A, Ion D, Andronic O (2023). Endometriosis as an uncommon cause of intestinal obstruction-a comprehensive literature review. J Clin Med.

[10] Allan Z (2018). A case of endometriosis causing acute large bowel obstruction. Int J Surg Case Rep.

[11] Hamdy O, Ragab D, Farouk B (2025). Endometriosis-induced acute large bowel obstruction and endometriotic deposits in pericolic lymph nodes: a case report. J Endometr Pelvic Pain Disord.

[12] Yong PJ, Bedaiwy MA, Alotaibi F, Anglesio MS (2021). Pathogenesis of bowel endometriosis. Best Pract Res Clin Obstet Gynaecol.

[13] Ragab MI, Altabba AM, Hilmi S, Attia KE, Elnogoomi AI (2023). Endometriosis causing large bowel obstruction: a case report. Cureus.

[14] Alexandrino G, Lourenço LC, Carvalho R, Sobrinho C, Horta DV, Reis J (2018). Endometriosis: a rare cause of large bowel obstruction. GE Port J Gastroenterol.

[15] Sung H, Siegel RL, Laversanne M (2025). Colorectal cancer incidence trends in younger versus older adults: an analysis of population-based cancer registry data. Lancet Oncol.

[16] Lee TH, Kim JS, Baek SJ, Kwak JM, Kim J (2023). Diagnostic accuracy of carcinoembryonic antigen (CEA) in detecting colorectal cancer recurrence depending on its preoperative level. J Gastrointest Surg.

